# Process-Driven Structural and Property Evolution in Laser Powder Bed Fusion of a Newly Developed AISI 316L Stainless Steel

**DOI:** 10.3390/ma18143343

**Published:** 2025-07-16

**Authors:** Amir Behjat, Morteza Shamanian, Fazlollah Sadeghi, Mohammad Hossein Mosallanejad, Abdollah Saboori

**Affiliations:** 1 Department of Materials Engineering, Isfahan University of Technology, Isfahan 84156-83111, Iran; amir.behjat@polito.it (A.B.); shamanian@cc.iut.ac.ir (M.S.); mh.mosallanejad@iut.ac.ir (M.H.M.); 2 Department of Management and Production Engineering, Politecnico di Torino, Corso Duca degli Abruzzi 24, 10129 Torino, Italy; 3 Integrated Additive Manufacturing Center (IAM@PoliTo), Politecnico di Torino, Corso Castelfidardo 51, 10129 Torino, Italy; 4 Graduate Institute of Ferrous and Energy Materials Technology, Pohang University of Science and Technology, Pohang 37673, Republic of Korea; fazlollah.sadeghihosnijeh@brunel.ac.uk; 5 Brunel Center for Advanced Solidification (BCAST), Brunel University, Middlesex, London UB8 3PH, UK

**Keywords:** Metal Additive Manufacturing, laser powder bed fusion, AISI 316L, new alloys, processability

## Abstract

The lack of new materials with desired processability and functional characteristics remains a challenge for metal additive manufacturing (AM). Therefore, in this work, a new promising AISI 316L-based alloy with better performance compared to the commercially available one is developed via the laser powder bed fusion (L-PBF) process. Moreover, establishing process–structure–properties linkages is a critical point that should be evaluated carefully before adding newly developed alloys into the AM market. Hence, the current study investigates the influences of various process parameters on the as-built quality and microstructure of the newly developed alloy. The results revealed that increasing laser energy density led to reduced porosity and surface roughness, likely due to enhanced melting and solidification. Microstructural analysis revealed a uniform distribution of copper within the austenite phase without forming any agglomeration or secondary phases. Electron backscatter diffraction analysis indicated a strong texture along the build direction with a gradual increase in Goss texture at higher energy densities. Grain boundary regions exhibited higher local misorientation and dislocation density. These findings suggest that changing the process parameters of the L-PBF process is a promising method for developing tailored microstructures and chemical compositions of commercially available AISI 316L stainless steel.

## 1. Introduction

Stainless steels are widely used in different industries, such as chemical production, food processing, surgical implants, and nuclear reactors, due to their popularity and versatility in equipment and facility construction [1,2,3]. In recent times, steel manufacturers and research institutions have been actively investigating and pushing forward the alloy design of stainless steels in order to achieve a combination of desirable qualities such as excellent biocompatibility, corrosion and wear resistance, as well as good strength and ductility [4,5,6]. In addition, the production of complex metal structures using traditional manufacturing techniques remains a challenge as it often involves extended production time, high costs, and possible reduced durability in some cases [7,8].

One of the approaches to overcome these issues could be using additive manufacturing (AM) techniques that offer numerous benefits, including significantly reducing the manufacturing cycle, minimizing waste, and lowering production costs [9,10]. Additionally, AM enables personalized manufacturing while embracing a digital, intelligent, and environmentally friendly production approach [11,12]. Metal AM technologies, such as the laser-based powder bed fusion process (L-PBF), also known as selective laser melting (SLM), have been used to process different alloys such as steels [13,14], nickel-based superalloys [15,16], zircunium [17,18], aluminum [19,20], titanium [21,22], copper [23,24], etc. During this process, a component is generated by selectively melting layers of powder on top of one another using a high-energy laser beam. It is well documented that the critical factors in the processing of materials using L-PBF include laser power, laser scanning speed, powder layer thickness, hatch distance, and the scanning strategy [12,20,25]. Therefore, when a new alloy is considered for the first time for the L-PBF process, its processability should be evaluated via a design of experiment approach so as to analyse its response to different combinations of process parameters. This procedure aims to find the optimum combination of process parameters to secure a productive and defect-free production. As far as the AISI 316L stainless steel is concerned, there are numerous documented instances of successful fabrication of AISI 316L parts through the L-PBF process, and no significant challenge has been reported [26,27,28]. Despite the easy processability of AISI 316L via L-PBF technology, new compositions based on this alloy are still needed to broaden its applications in different sectors. Hence, research in AM of AISI 316L stainless steels is motivated by the increasing focus on alloy design to improve the functionality of AISI 316L-based parts. Various process strategies have been reported, including altering the shield gas environment [29,30], producing functionally graded materials [31,32], and modifying the feedstock by adding small amounts of metallic elements or intermetallic compounds to the initial powder [4,33,34,35]. Selecting suitable additives to improve the microstructure of steel and prevent the formation of harmful inclusions may improve both mechanical strength and resistance to corrosion [34,36]. Notable cases of successfully modified stainless steels with the addition of titanium (Ti) [37], silver (Ag) [38], tungsten (W) [39], titanium-nitride (TiN) [40], titanium carbide (TiC) [41], cerium oxide (CeO_2_) [42], and chromium nitride (CrN) [43] have been reported. However, it is reported that adding copper (Cu) to stainless steel can enhance its mechanical properties, corrosion resistance and antibacterial properties [44,45,46]. It has been reported that the variation in mechanical performance among these alloys may be attributed to factors such as the copper content, the method of fabrication, and the post-heat treatment processes applied to the samples [14].

Wang et al. produced a porous scaffold made of AISI 316L-4.5Cu with low stiffness using L-PBF technology. This scaffold exhibited stiffness similar to bone, which is desirable for avoiding stress shielding. According to their work, adding Cu did not significantly impact the strength and stiffness of the AISI 316L scaffold [47]. Liu et al. utilized Directed Energy Deposition (DED) to coat copper on AISI 316L stainless steel with varying levels of Cu content. Their research revealed that adding Cu can refine the austenite dendritic structure and that increasing the copper content can enhance the corrosion resistance of the coatings [48]. In another work, Mirzababaei et al. fabricated a AISI 316L-copper composite with different volume ratios utilizing the L-PBF method. They reported 2.5 times thermal conductivity enhancement by adding up to 60 vol.% copper since the continuous network of the copper phase is formed throughout the stainless steel matrix [49]. Lately, the results from our previous works showed that in situ alloying AISI 316L with copper element led to decreased grain size, better combination of strength and elongation, and enhanced antibacterial properties against *S. aureus* and *E. coli* bacteria in comparison with AISI 316L printed by the L-PBF process [14,50].

Despite all these previous works, no work in the literature has systematically investigated the development of AISI 316L-Cu and evaluated the influence of process parameters on microstructure, relative density, and surface quality of the as-built AISI 316L-Cu stainless steel components. The results of this study certainly provide new directions for further development of austenitic stainless steels in different industries, such as medical applications, through AM in situ alloying techniques.

## 2. Materials and Methods

**Figure 1:**
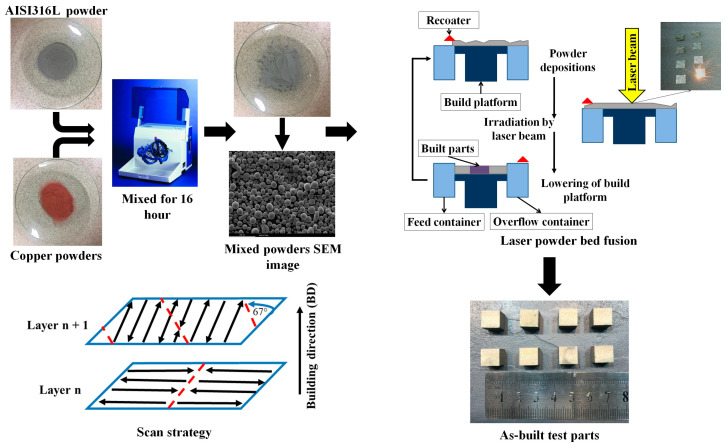
A schematic of workflow for producing as-built AISI 316L-Cu samples via the L-PBF process.

Figure 1 provides an overview of the steps involved in producing the specimens, from the initial powder preparation to the sample production via the L-PBF process. The details of each step are explained in the following subsections.

### 2.1. Feedstock Preparation

In this research, feedstock materials were prepared by mixing gas-atomized, spherical AISI 316L powder, with a particle size range of 10–45 μm (Oerlikon, Pfäffikon, Switzerland), with gas-atomized, spherical, pure Cu powder (>8 µm, 99% purity, Sandvik Osprey Ltd., Stockholm, Sweden). The mixing was performed through ball-milling for 16 h. The combination of powders exhibited excellent flow characteristics, and the composition is detailed in Table 1. The morphology of the mixed powder particles was examined using scanning electron microscopy (SEM, Philips XL, Amsterdam, The Netherlands) equipped with an energy-dispersive X-ray spectrometer (EDS).

### 2.2. Sample Production

Eight cubic samples of 10 × 10 × 10 mm^3^ were produced using a Concept Laser Mlab Cusing-R machine (Lichtenfels, Germany) equipped with a 100 W fiber laser. The manufacturing process was conducted under an Ar environment with oxygen below 2000 ppm. The layer thickness was set to 25 µm, and a strip 67° build strategy was employed, with the contour being scanned ahead for each layer.

The process parameters employed for the fabrication of various AISI 316L-Cu samples are detailed in Table 2, with the Volumetric Energy Density (VED) calculated according to Equation (1):

E = P/(v × h × t)(1)

where P represents the laser power (W), v is the laser scan rate (mm/s), h is the distance between hatch lines (mm), and t is the thickness of each layer (mm). Once the cubic samples were built, a wire electrical discharge machine (WEDM) was utilized to remove the as-built cubes from the building platform.

### 2.3. Materials Characterization

The optimal processing parameters for AISI 316L-Cu were identified by evaluating the surface roughness and relative density of the as-built cubes. The average roughness (Ra) was assessed by taking three measurements at various points on the surface of each sample using a MarSurf RTP80 tester (Göttingen, Germany) following the ISO 428/JIS 80601 standard [51].

The relative density of each sample was calculated using the Archimedes principle. This involves measuring the weight of each sample when dry and then when submerged in a beaker of water at a constant temperature of 25 °C with a density of 0.9975 g/cm^3^. To find the density of the as-built specimens, the mass of each sample in water is divided by the volume of water displaced. The relative density is expressed as a percentage of the nominal density of AM AISI 316L, which is 7.99 g/cm^3^. Moreover, to evaluate the porosity content, type, and as-built microstructure, the center of each as-built sample (along the build direction (BD)) was cut, mounted, ground, and then polished following the standard procedure reported elsewhere.

The phase composition of the samples was identified by X-ray diffraction (XRD, Phillips, Amsterdam, The Netherlands) analysis with Mo Kα X-Ray target (0.7093 Å) radiation at 40 kV and 30 mA. XRD scans were conducted between 15° and 40° (2θ). For electron backscatter diffraction (EBSD) analysis, samples were polished mechanically until 0.05 colloidal suspension. Electropolishing was conducted in a solution of 20% perchloric acid using ethanol b LectroPol-5 (Struers, Ballerup, Denmark) to remove the effect of residual strain due to mechanical polishing. For microstructural analysis, a scanning electron microscope (SEM) (JEOL model JSM-7900F, Tokyo, Japan) was used at an accelerating voltage of 20 kV and 15 nA probe current to obtain EBSD data. The EBSD results were extracted using Oxford Aztec v6.0 and Aztec Crystal v2.0. The Vickers microhardness of each sample was determined using an MH4 Vickers hardness tester from Koopa, Sari, Iran. A test force of 300 g was applied to the top surface and cross-section of each sample with a dwell time of 15 s. The average microhardness was obtained by measuring 5 distinct points.

## 3. Results and Discussion

### 3.1. Powder Characterization

**Figure 2:**
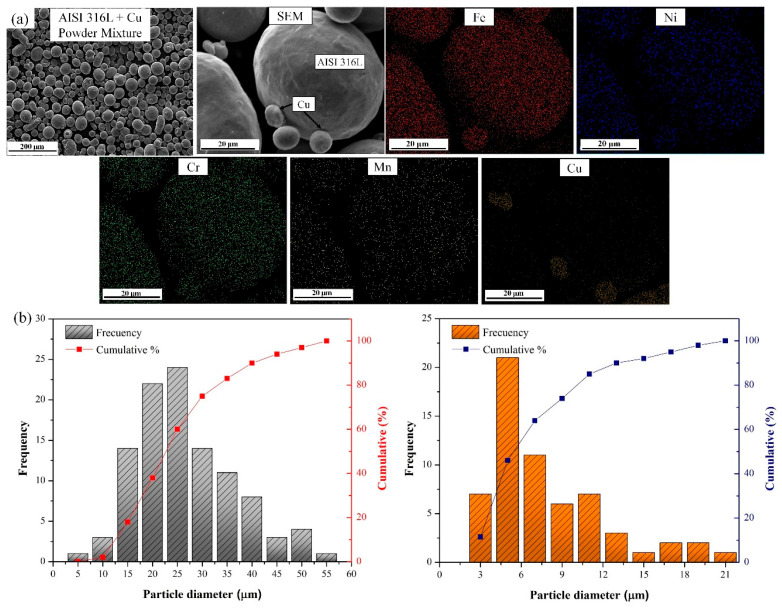
(**a**) Morphology and high magnification images of the powder mixture with corresponding EDS elemental maps, and (**b**) particle size distributions histograms of AISI 316L and Cu powders.

The morphology of the AISI 316L-Cu powder mixture is shown in Figure 2a. As can be seen in this figure, most of the starting powder particles exhibit spherical shapes with a homogenous distribution of the constituents. The EDS map analysis of the blended powders reveals a uniform distribution of copper particles within the AISI 316L matrix powder. This result confirms that the fine copper particles were positioned as satellites around the AISI 316L particles, ensuring the homogenous distribution of copper inside the AISI 316L matrix after printing. The particle size distributions of the starting powders were analysed following the image analysis approach using five figures per composition, and the resulting histograms are reported in Figure 2b. The histograms indicate that the average particle size of the starting AISI 316L and Cu powders is 27 µm (d10 = 13 µm, d50 = 23 µm, d90 = 40 µm) and 6.3 µm (d10 = 3.1 µm, d50 = 5.3 µm, d90 = 13.1 µm), respectively.

### 3.2. As-Built Quality Evaluation

**Figure 3:**
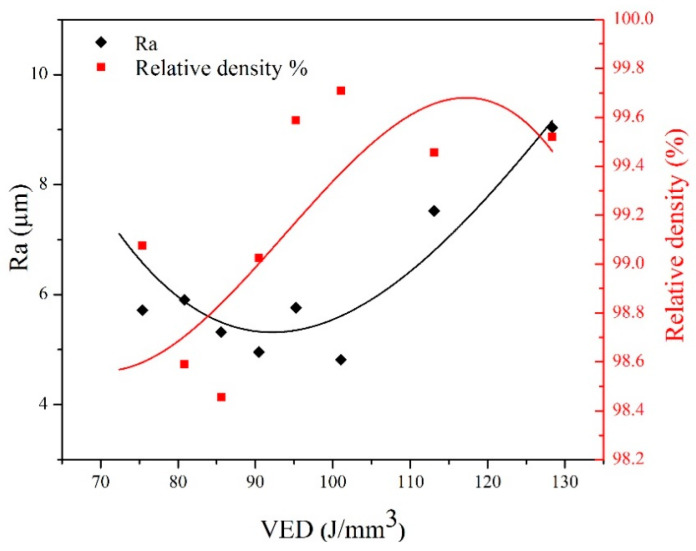
Surface roughness and relative density of the as-built AISI 316L-Cu samples as a function of VED.

The existing literature has firmly proven that different combinations of process parameters in the L-PBF process can lead to significantly different outcomes regarding defect content and surface quality [26,52]. Figure 3 shows the variation in the surface roughness and relative density of the as-built AISI 316L-Cu samples as a function of VED. This graph clearly confirms what has been reported in the literature regarding the influence of the VED on the as-built quality of the AISI 316L samples produced via L-PBF.

Two regions are considered for surface roughness and three for relative density of the as-built AISI 316L-Cu samples, as shown in Figure 3. The surface roughness of parts produced through AM is a significant parameter that plays a crucial role in defining their mechanical properties and susceptibility to corrosion. Compared to conventional manufacturing techniques, AM parts typically exhibit higher surface roughness, a characteristic closely linked to the VED utilized during manufacturing [53,54]. Thus, in this study, when the VED is less than 110 J/mm^3^ (region I), the as-built parts are in the successfully fabricated region. In this region, increased VED can help create a steady molten pool with beneficial surface tension and wetting properties, allowing the metal powder to melt and form a continuous and smooth surface. The as-built parts were in the high superheat region when the VED was higher than 110 J/mm^3^ (region II). In this region, an overabundance of heat production may adversely affect surface roughness due to the disturbance of the melt pool and the increase in recoil pressure [52]. Consequently, it can be inferred that the specimens fabricated with VED values of 101 J/mm^3^ and 128 J/mm^3^ exhibit the lowest and highest surface roughness, respectively.

**Figure 4:**
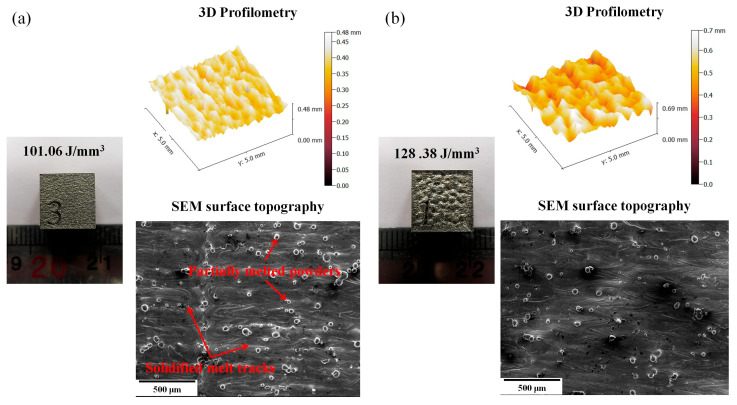
The surface topography analysis of the as-built AISI 316L-Cu samples produced using VED (**a**) 101 J/mm^3^ and (**b**) 128 J/mm^3^.

The optical micrographs, top surface topography, and 3D profilometry of the as-built AISI 316L-Cu samples at two different laser energy densities are shown in Figure 4. As can be seen in Figure 4a, which is related to the sample printed using 101 J/mm^3^, a smooth melted surface with partially melted powders were formed, and the presence of solidified melt tracks confirms the suitability of the laser energy density. However, as the VED energy was increased (Figure 4b), the liquid track flow increased dramatically, and splashing occurred, resulting in a rough surface. These observations suggest that an over-melting phenomenon and burning of metal powder are responsible for the increase in surface roughness.

It is well documented that as a powder-based fabrication technique, L-PBF inherently introduces porosity in the manufactured components, potentially influencing their mechanical characteristics and corrosion resistance [53,55,56,57]. In general, the porosity present in the as-built AM parts can be classified into two main types based on their geometry: regular or spherical pores, and irregular or Lack of Fusion (LOF) pores. The mechanism of pore formation during the AM processes has been extensively studied and investigated [58]. According to previous reports, the porosity type in the as-built parts depends on several factors, such as the process parameters and powder quality. Therefore, it is reported that by using appropriate process parameters, it is possible to optimize the density and change the porosity types in the final part [26,58,59].

As illustrated in Figure 3, a low VED where the laser energy density is not sufficient to melt the complete powder layer can significantly expedite the development of pores due to the presence of unmelted powder and inadequate melting of the powder bed. Irregular/LOF pores are typically situated near interlayer or hatch boundaries and can significantly reduce the density of the as-built parts [60]. These pores can considerably impact the formation and spread of pits and cracks in the as-built parts. Generally, the relative density and surface quality of as-built parts are poor in this region. By increasing the VED values from 90 J/mm^3^ to 110 J/mm^3^, melt pools with a relatively higher temperature and of a larger size were established, which eased liquid flow to fill the pores. Cherry et al. found that an energy density of approximately 104 J/mm^3^ is necessary to achieve a negligible porosity fraction in the L-PBF of AISI 316L [26]. Nevertheless, in the case of specimens produced with high VED, an unstable and irregular molten pool is formed during the L-PBF procedure and is identified as a factor that promotes the entrapment of gases within the molten material as the molten material solidifies. Pores are commonly observed near the unmelted powder particles or due to the entrapment of gas during the initial processing stages of the powder or melt pool, for instance, during gas atomization or laser melting processes. Additionally, the destabilization of the melt flow by the Marangoni force can enhance the likelihood of pore formation. Numerous quantitative investigations have been conducted to support these findings [52,61].

**Figure 5:**
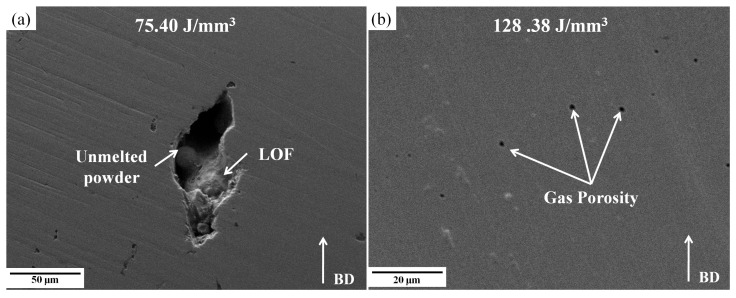
SEM micrographs of the as-polished surfaces of the as-built AISI 316L-Cu samples, indicating the formation of (**a**) LOF/irregular-shaped, and (**b**) spherical/regular-shaped pores.

A microscope analysis was conducted to investigate porosity formation in more detail. Figure 5 illustrates the two distinct types of pores observed during the L-PBF process to produce AISI 316L-Cu samples printed using VED values of 75 J/mm^3^ and 128 J/mm^3^.

As mentioned earlier, when the VED is low (75 J/mm^3^), the pores are interconnected and take on an irregular shape. In this instance, the porosity is defined by significant cavities, as illustrated in Figure 5a, which may reach sizes of up to 130 ± 23 µm and contain loosely bound particles measuring 28 ± 11 µm. This indicates that these particles are likely not completely melted powder particles. One potential rationale for this phenomenon is that when the laser energy density is low, the melt pool size is decreased [62], resulting in insufficient melting of the powder particles to facilitate adequate bonding between the layers. However, when the VED is increased (128 J/mm^3^), the pores become mostly spherical (Figure 5b). As reported, the larger melt pools formed at elevated VEDs may also exhibit a higher susceptibility to the formation of solidification micro-shrinkage porosity [38]. Therefore, after examining surface roughness and relative density, this research has determined that a VED value of 101 J/mm^3^ is optimal for attaining a satisfactory quality of new AISI 316L-Cu alloy fabricated via L-PBF.

**Figure 6:**
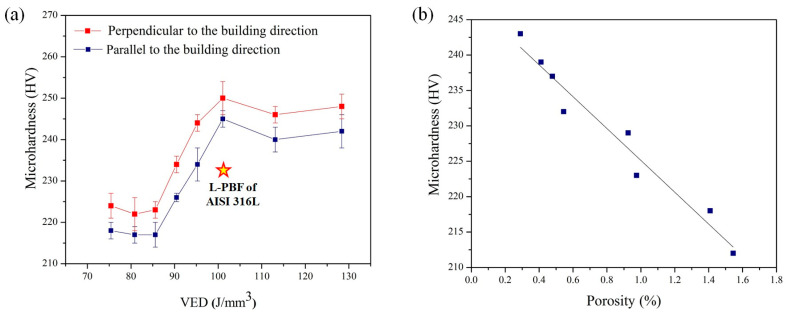
Microhardness curves of the as-built AISI 316L-Cu samples as a function of (**a**) VED and (**b**) porosity.

Figure 6a illustrates the mean microhardness of AISI 316L-Cu alloy specimens fabricated via L-PBF with varying VED values in orientations perpendicular and parallel to the BD. The results from the hardness tests indicate a correlation with the porosity results obtained from varying VED input (Figure 6b). Specifically, an increase in porosity was associated with a decrease in hardness. As a matter of fact, porosities emerged as the vulnerable areas of the material when subjected to stress, resulting in a reduction in microhardness. Therefore, the results indicate that the microhardness of the sample fabricated with a VED value of 101 J/mm^3^ and a density of 99.81% was higher than that of the sample fabricated with a VED value of 85 J/mm^3^ and a density of 98.44%. Additionally, the average microhardness of the AISI 316L-Cu alloys in this study was found to be higher than that of AISI 316L produced using L-PBF, which had a reported microhardness of 228 ± 7 HV [26,50]. The increased microhardness can be associated with the enhanced solid solution strengthening capability of the Cu element in the AISI 316L alloy [44]. The varying atomic radii of alloy components may result in lattice distortion, thereby improving the solid solution strengthening effect [48].

### 3.3. Microstructural Characterization

**Figure 7:**
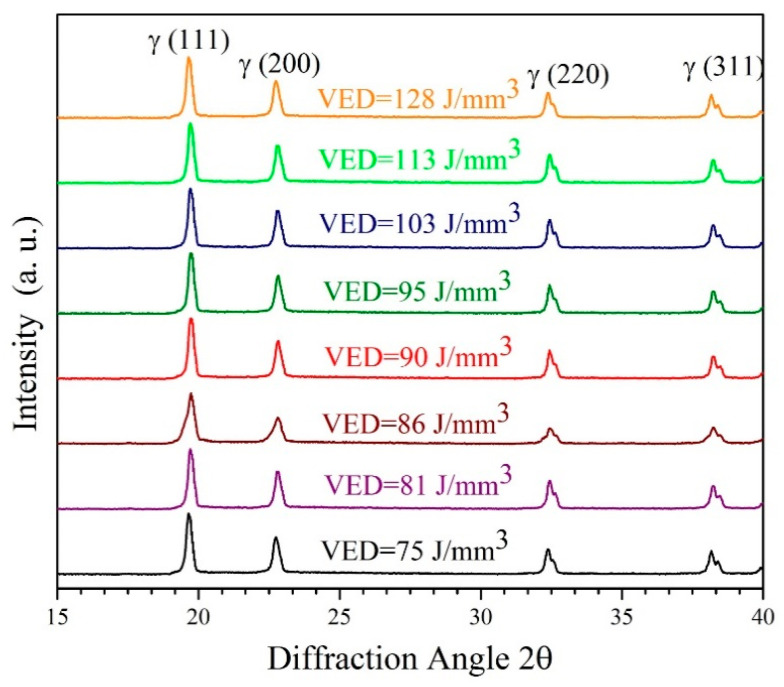
XRD patterns on sections perpendicular to the BD of the as-built AISI 316L-Cu samples.

To investigate the impact of process parameters on the solid solution of copper in austenite, XRD analysis was conducted for the as-built samples produced using different VED values, and the outcomes are presented in Figure 7.

As can be seen, the only phase consistently observed across all the samples is the austenite phase, with no detection of any phases containing copper or undesirable phases such as sigma phase (σ) and martensite. Indeed, as illustrated in Figure 7, the (111) plane of the austenite structure exhibits the highest peak intensity ratio (I/I_max_) among atomic planes, with minimal variations in relative intensity observed when comparing spectra of different samples. It has been noted that altering the VED results in modifications to the temperature gradient and grain growth rate in the solidification process, consequently impacting the structural formation. Furthermore, the presence of copper can modify the solidification temperature range and affect the solidification rate. Copper has a lower melting point compared to stainless steel, so it can influence the solidification temperature and potentially lead to eutectic reactions or the precipitation of copper-rich phases, the latter of has not been detected in this study.

Furthermore, the analysis of crystal size, microstrain and dislocation density from the XRD spectra was conducted through application of the Williamson–Hall formula, which is shown below [63]:

(2) $$\beta_{hkl}\cos\theta =\frac{K\lambda }{D}+4\varepsilon \sin\theta$$

**Figure 8:**
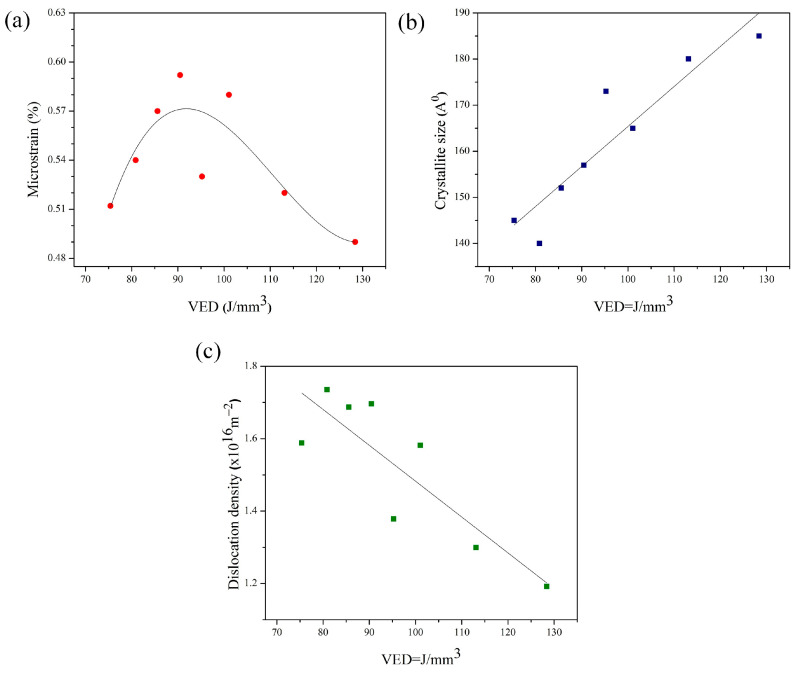
Variations in (**a**) crystallite size, (**b**) lattice microstrain, and (**c**) dislocation density as a function of VED.

Here, K = 0.94 is a shape factor, λ = 0.7093 Å nm (wavelength of Mo Kα), and β is the peak width at half maximum. D, ε and θ are crystallite size in nm, microstrain and Bragg peak angle, respectively. The equation employed for assessing dislocation density through the parameters of crystallite size and microstrain is expressed as follows:

(3) $$\rho_{hkl}=\frac{2\sqrt{3}\varepsilon }{D_{hkl}b}$$

where b is the Burgers vector for an FCC structure. Figure 8a–c plots the variations in these microstructure features against VED values.

Figure 8a demonstrates the relationship between the crystallite size of the austenite phase and the VED. The data indicates a progressive growth in crystallite size with increasing VED. This phenomenon can be explained by the fact that at the lower VED values, dendrites do not have sufficient time to develop due to the rapid cooling rate. Furthermore, higher VEDs lead to an increased size of the melt pool, resulting in larger crystallite sizes. These findings are consistent with prior research [64]. The evolution of microstrain corresponding to different VED levels is illustrated in Figure 8b. This behavior can be attributed to two primary phenomena. Firstly, the development of internal residual stresses is associated with the non-equilibrium solidification resulting from the comparatively rapid cooling rate experienced during the printing process [65]. Therefore, an increase in VED results in a decrease in the cooling rate and an increase in maximum temperature, subsequently increasing the diffusion rate. This increased diffusion rate leads to a more uniform distribution of copper throughout the metal matrix, resulting in a slightly larger lattice distortion. Secondly, higher VED values result in larger melt pool sizes and longer exposure times to high temperatures, which provide more time for dislocation rearrangement and annihilation, ultimately leading to less lattice distortion. This result was consistent with the dislocation density versus VED values shown in Figure 8c. It is worth mentioning that Cu can effectively increase the stacking faults energy (SFE) of AISI 316L austenitic steel, reduce the width of partial dislocations within the steel, and consequently facilitate the combination of partial dislocations and the formation of perfect dislocations [66].

**Figure 9:**
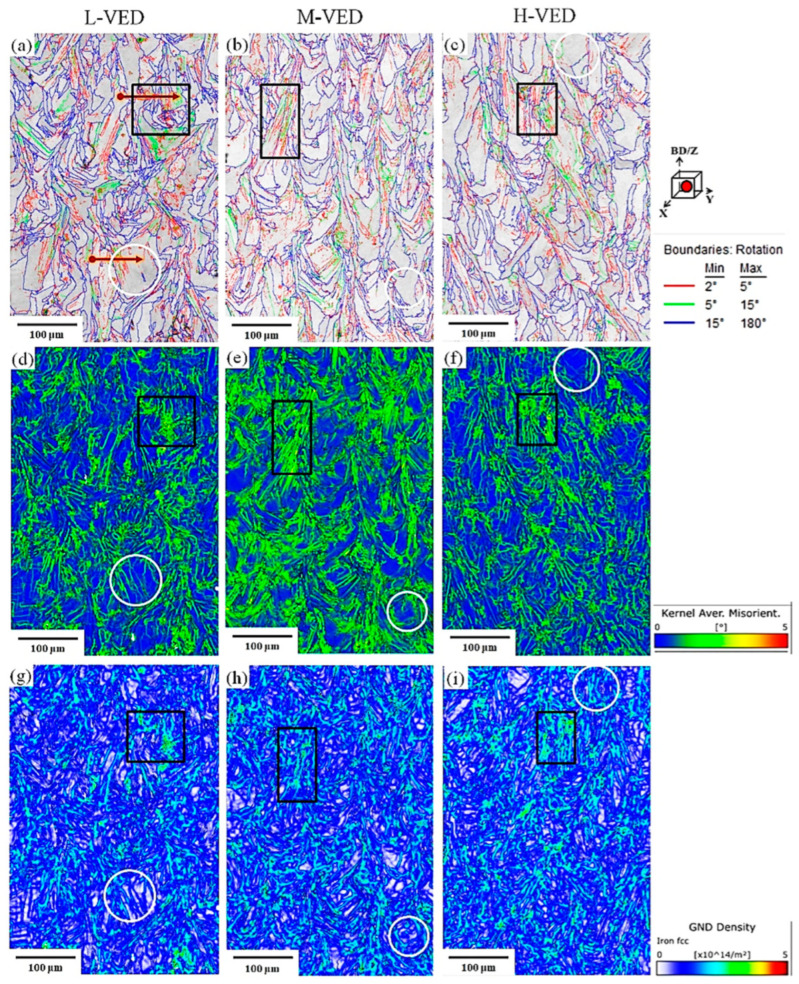
(**a**–**c**) Image quality with grain boundaries, (**d**–**f**) KAM results, and (**g**–**i**) GND maps for the as-built AISI 316L-Cu samples. (Red arrows: misorientation angle path, black rectangular box: high stored energy regions, and white circles: low stored energy regions).

**Figure 10:**
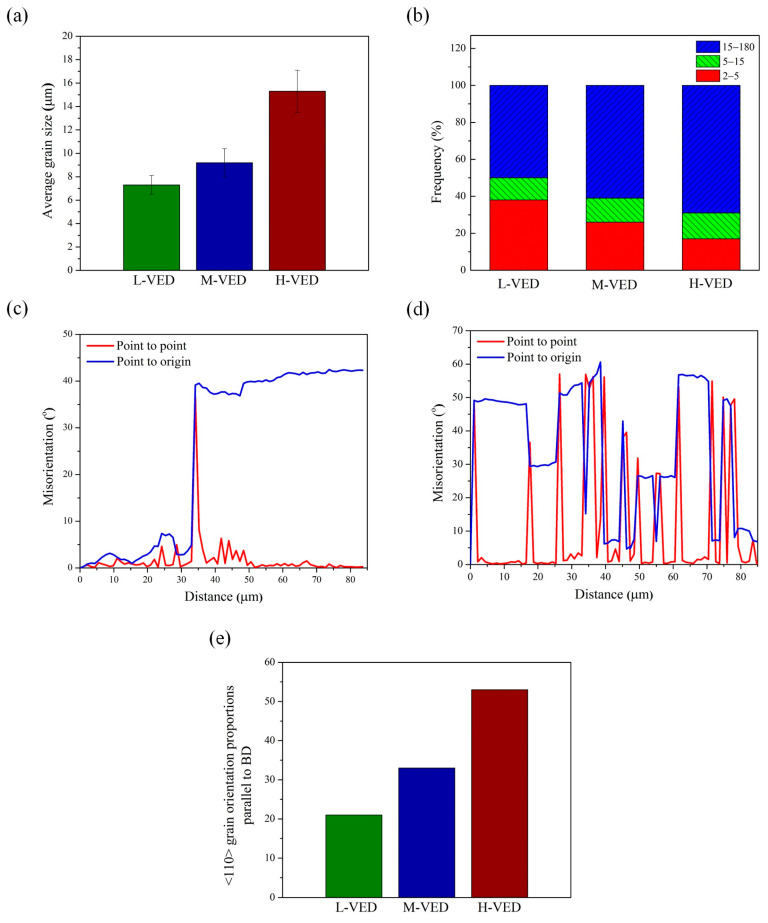
(**a**) Average grain size, (**b**) fraction of LAGBs and HAGBs, (**c**,**d**) misorientation angle plot for the red arrows shown in Figure 9a, and (**e**) <110> grain orientation proportion parallel to the BD.

**Figure 11:**
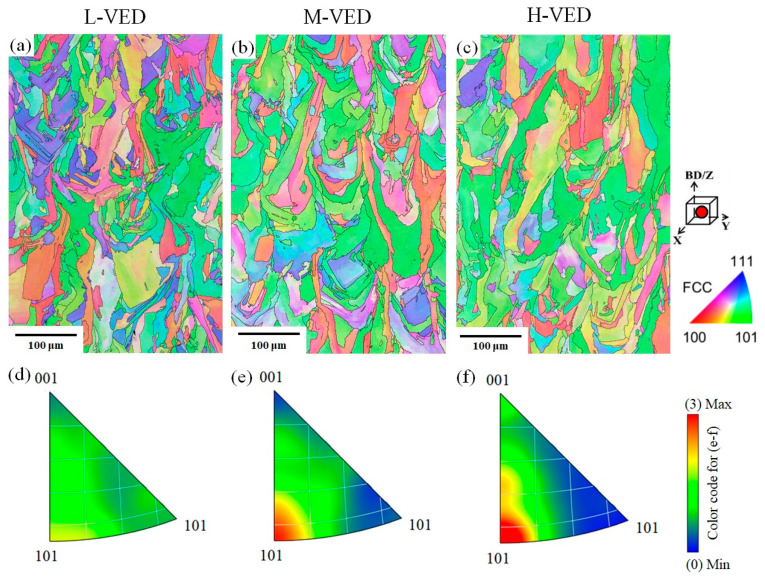
(**a**–**c**) colour-coded IPF maps, and (**d**–**f**) the corresponding IPF at the Z-axis (i.e., BD) as a function of the VED values.

Furthermore, to establish the link between VED values and the microstructure evolution during printing, EBSD analyses were performed on three samples. The EBSD band contrast (BC) maps with the grain boundary distribution, inverse pole figure (IPF) maps, kernel average misorientation (KAM) maps, geometrical necessary dislocations (GND) maps, and statistical analysis of EBSD data of three different specimens are presented in Figure 9, Figure 10 and Figure 11. The characterized samples had VED values of 85, 101 and 113 J/mm^3^, which are labelled throughout the text as Low (L-VED), Medium (M-VED) and High (H-VED), respectively. The Z-axis of all images in Figure 9 corresponds to the build direction.

The grain boundary colouring distribution in Figure 9a–c was divided into high angle 15° ≤ GB, low angle 5° ≤ GB ≤ 15°, and 2° ≤ GB ≤ 5° in black, green, and blue, respectively. The average grain size and the fraction of low-angle grain boundaries (LAGBs), high-angle grain boundaries (HAGBs), and misorientation angle plots of the samples were statistically calculated from the Figure 9 data and plotted in Figure 10a–d.

The results show that the grain size of the L-VED, M-VED and H-VED samples was 7.3 ± 1.4, 9.2 ± 2.1 and 15.3 ± 2.7 µm, respectively (Figure 10a), which aligns with reports in the literature [67]. It is well reported that L-PBF-produced AISI 316L typically exhibits complex microstructures characterized by cellular sub-grains within grain structures. Based on a comparison of the grain size between the L-PBF of AISI 316L-Cu in this study and that of AISI 316L in previous studies [50,68], it can be inferred that the addition of copper increased the cooling rate, which consequently results in grain refinement. Indeed, this increase in cooling rate is believed to be due to the higher thermal conductivity of copper compared to AISI 316L. Therefore, by controlling the cooling rate by adding appropriate elements during AM, it is possible to tailor the microstructure and mechanical properties of materials to meet specific requirements. The grain boundary fraction of the as-built AISI 316L-Cu samples in Figure 10b reveals that in H-VED, the HAGB fraction is 69% while the LAGB fraction is 31%.

In contrast, in L-VED, about 50% of the boundaries are LABs, and about 50% are HABs. It is well known that the existence of more LAGBs in L-VED can be attributed to the rapid cooling of molten material, which is caused by strain gradient and dislocations in the samples [69,70,71,72]. Moreover, this difference in microstructural characteristics can be ascribed to the low cooling rate and high temperature experienced for longer time intervals in the H-VED sample, which causes recrystallization, grain growth, and annihilation of the LAGBs, turning them into HAGBs.

The distribution of local microstrain and dislocation density in samples can be extracted from KAM and GND maps, respectively. The KAM parameter is considered equivalent to the amount of microstrain and stored energy inside the structure. As shown in Figure 9d–f, there are colour changes in different parts of KAM maps for all samples. It can be noticed that the grain boundary regions have more local misorientation and dislocation density than the interior of the grains. Moreover, the KAM maps show that the amount of microstrain inside the structure of the L-VED sample is slightly higher and non-uniformly distributed in the structure.

A relationship between the grain boundaries, KAM, and GND results can be observed, specifically when comparing the black rectangular box regions for each sample. This indicates that areas with high stored energy exhibit a greater concentration of misorientation boundaries. This phenomenon can be interpreted in connection with GND maps (Figure 9g–i), as a greater dislocation density is necessary to adjust the misorientations. Indeed, differential growth directions and rates of columnar crystals lead to the emergence of substantial boundary stresses, which in turn, give rise to numerous dislocations in grain boundaries and melt pool boundaries. Additionally, there were regions of low stored energy, encompassed with white encircled regions for each sample. There is a slight orientation variation within these areas, represented by low misorientation variations, which usually accommodate relatively less energy and low GND density. Furthermore, the results are supported by the linear profiles of misorientations, extracted along the red arrows in Figure 9a. These misorientation plots indicate that the region with high KAM and GND values has fluctuating misorientations (Figure 10c). In contrast, Figure 10d illustrates the area with minor misorientation variations is essentially consistent, and the local microstrain inside the grains is small. Therefore, all samples showed an overall heterogeneous distribution of stored energy. Comparing this trend with the XRD analysis of Figure 8b reveals that a local microstrain is less proportional to the VED values.

The texture development during the L-PBF process is dictated by the direction of heat flow, thermal gradient, and the specific nucleation conditions during solidification [59,73,74,75,76]. These items influence the alignment of the growth direction (precisely the (100) direction for cubic materials) with the build direction and the most conducive conditions for promoting epitaxial growth. Indeed, the (100) crystallographic orientation is a favorable growth direction in FCC alloys due to its notably open structure, which facilitates enhanced diffusivity along this axis [76]. However, the final growth orientation, which determines the epitaxial texture, is contingent upon the degree of misalignment between the growth direction of <100> and the build direction, along with the thermal gradient value [77].

It is widely recognized that these characteristics can be adjusted by modifying the main process parameters of the L-PBF process, including laser beam power, scanning strategy, powder feedstock properties, and build plate temperature [56,78,79]. Sun et al. achieved a single-crystalline-like structure in AISI 316L stainless steel through the X-scan strategy and high energy density application via L-PBF of AISI 316L alloy [80]. Moreover, as reported in previous works, the alteration of scan strategy is regarded as a technique to reduce the crystallographic texture in L-PBF-produced AISI 316L alloy. This is achieved by introducing randomness to the thermal gradient during printing, which hinders the preferential growth along a specific <100> crystallographic direction of nuclei [69,81].

Overall, based on the IPF maps illustrated in Figure 11a–c and the data derived from the ratio of grains exhibiting (110) orientation parallel to the BD texture, as shown in Figure 10e, it can be concluded that increasing VED values lead to a notable enhancement of the austenite (110) texture along the BD. The corresponding IPF figures (Figure 11d–f) also confirm the enhanced micro-texture of (110) ∥ BD for the H-VED sample. This condition creates a localized heat accumulation and a larger melt pool size that can result in homogeneous melting of new powder. In contrast to the H-VED sample, the M-VED and L-VED samples exhibited much weaker textures, which were close to a random texture. Additionally, as illustrated by the IPF contours in Figure 11a–c, grains with varying orientations are evident in these two samples. Thus, the findings suggest that utilizing the strip 67° build strategy pattern, which induced the generation of a complex thermal gradient in combination with a low-to-medium VED value, resulted in fairly isotropic textures and microstructures.

During the metal AM process, the melt pool solidification occurs directionally from the melt pool boundary toward the top center of the melt pool, i.e., the heat gradient. This behavior, coupled with epitaxial growth, leads to columnar grains usually spanning across multiple build layers. Meanwhile, melt pool shape, dimensions, and thus, the overlapping, which largely determines the thermal gradient direction (i.e., normal to the melt pool edge), can largely influence the microstructure evolution and texture formation during rapid solidification by altering the solid/liquid interface migration. It can also be mentioned that various configurations of molten pools, such as convection and keyhole modes, are influenced by the amount of energy exerted during the melting process, which depends on the process parameters. This phenomenon has been employed in other works [44,45,46,47] to tailor the microstructure and the mechanical properties of metal components produced by AM. Therefore, to better understand the melt pool geometrical changes, the melt pool geometrical evolution, specifically the melt pool aspect ratio, i.e., *R* = depth/width, should be investigated. Using the reported scaling laws for the L-PBF of metals, Coen et al. [82] offered a methodology to account for the absolute melt pool dimensions, including *R*. Meanwhile, in this work, the L-VED, M-VED, and H-VED samples were produced using P = 95 W, with the scanning velocity being 600, 500, and 400 mm/s, respectively. These process parameters are closely matched with those predicted and tested by Coen et al. [82], for which the melt pool aspect ratio was reported to be 0.98, 1.12, 1.31.

**Figure 12:**
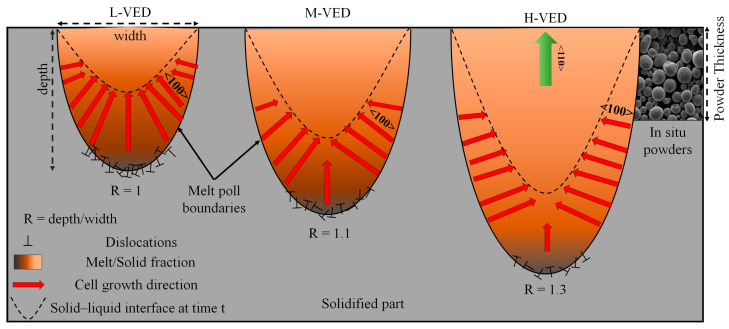
Schematic illustration of melt pool geometrical and microstructure evolution with VED variations.

Based on the above discussion, schematic diagrams of melt pool shape and microstructure features (crystal growth direction and dislocations) under different VEDs are shown in Figure 12. At L-VED, the melt pool has a low aspect ratio, leading to the inward growth of columnar crystals following melting, which subsequently transition gradually towards the BD and are followed by grains with random texture. On the one hand, high densities of crystal defects like grain boundaries and dislocations can be generated by other processes besides melt pool boundaries. It is possible that numerous dislocations are produced due to the competitive growth between the columnar crystals growing from the side area of the melt pool and those growing preferentially in the center of the molten pool. On the other hand, within the H-VED sample, an enhancement was observed in grains exhibiting a (110) ∥ BD texture component. This enhancement may be attributed to the predominant lateral movement of the solid–liquid interface. Consequently, when the bottom–center thermal gradient is weaker than the side–center gradient, there is minimal opportunity for cellular growth along the BD at the bottom of the melt pool. Indeed, when cells undergo vertical development, their growth is hindered by the lateral movement of the solid–liquid interface.

**Figure 13:**
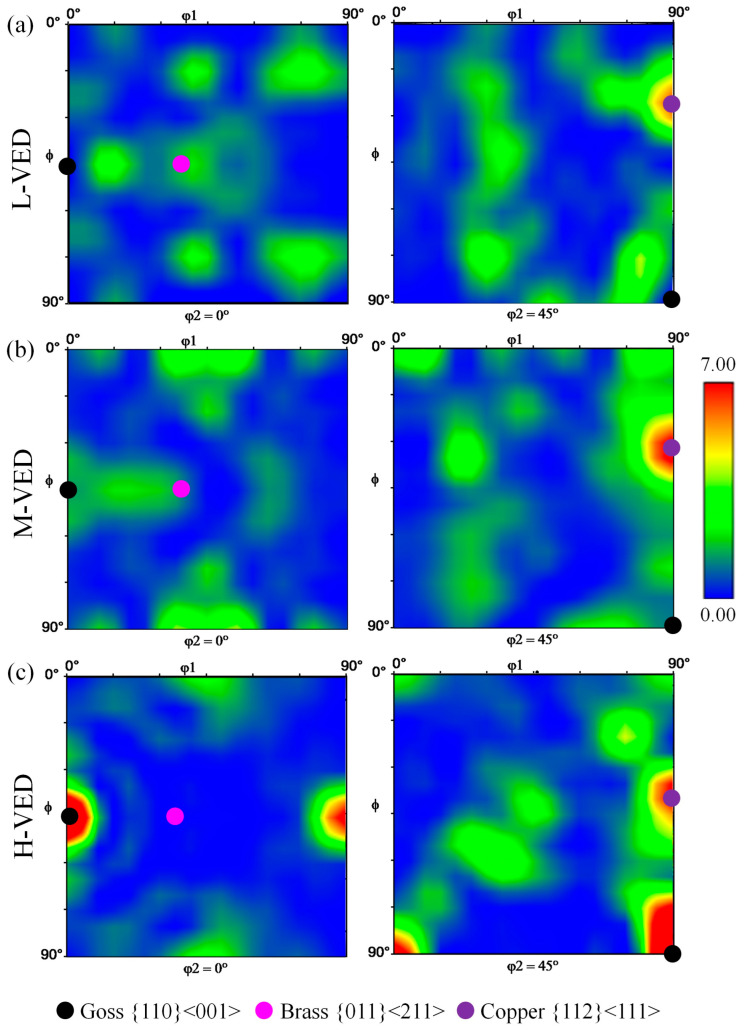
Comparison of constant sections (φ2 = 0° and 45°) of the ODF in Euler space for (**a**) L-VED, (**b**) M-VED, and (**c**) H-VED.

Furthermore, in order to examine the influence of VED values on the development of texture components, Figure 13 depicts the sections of the orientation distribution function (ODF) at Φ2 = 0 and 45° for three samples obtained from the EBSD data. The L-PBF process involves layer-by-layer melting and solidification, which can lead to the development of specific crystallographic orientations. The rapid cooling rates and thermal gradients inherent to this process favor the growth of grains in certain directions, resulting in preferred orientations like Goss, Copper, and Brass. The interplay between the cooling rates, scan strategies, and powder characteristics can significantly influence the final texture [83,84].

As documented, the Goss, Copper, and Brass textures represent significant microstructural features in stainless steel, particularly as they are affected by the parameters of L-PBF. The Goss texture, identified by the {110} crystallographic orientation, contributes to enhanced ductility and formability, although it may also lead to anisotropic behavior. Conversely, the Copper texture, characterized by the {112} orientation, is associated with improved strength and toughness, rendering it beneficial for applications subjected to high stress. The Brass texture, which also exhibits the {110} orientation, provides a favorable compromise between strength and ductility, making it suitable for a diverse range of applications [85].

As illustrated in Figure 13a–c, it was observed that all specimens displayed a Copper {111} <112> texture regardless of the conditions (purple spots). Furthermore, a comparison of the ODF data shows an interesting trend in reducing Brass texture {011} <211> (pink spots) and enhancing Goss texture {110} <001> (black spots) from L to M and H conditions. Indeed, the formation of a rather columnar grain arrangement in the H-VED specimen led to a partial texture linked to the Goss and Copper orientations. According to previous studies, laser power is the primary processing parameter that affects the texture of the material. Specifically, high laser powers have been found to result in the development of strong Goss textures [73,79,86]. Apart from the laser power, as mentioned before, the scanning strategy also plays an important role in controlling the texture of the material. By introducing rotation between layers, the epitaxial growth of grains can be weakened, which promotes the nucleation of finer grains with different crystallographic orientations. This can lead to weaker overall crystallographic textures, as opposed to strong epitaxial growth [78,87]. Conversely, a recent study conducted by Chen et al. indicated that in the L-PBF of AISI 316L, the hatching distance emerged as the most critical process parameter influencing texture [88]. This is attributed to its direct effect on the arrangement of melt pools and the inter-track overlap ratio. The researchers determined that an overlap ratio below 38% encourages the formation of polycrystalline microstructures, while ratios ranging from 38% to 53% promote <101> ∥ BD textures, and ratios exceeding 53% result in <100> ∥ BD textures. In contrast, laser power and scanning speed exert an indirect influence on the overlap ratio by modifying the size of the melt pool, with their impact diminishing at elevated laser energy densities. These conclusions were corroborated through both simulations and experimental validation, thereby establishing a dependable framework for texture control [88].

**Figure 14:**
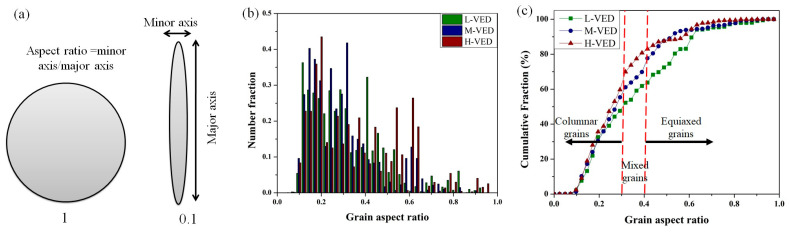
(**a**) Schematic representation of major and minor axes and the aspect ratio, and (**b**,**c**) the impact of varying VED values on the proportion of equiaxed and columnar grains.

Furthermore, to examine the impact of VED values on grain morphology, the proportion of equiaxed and columnar grains was assessed, as illustrated in Figure 14. The morphology of grains is predominantly determined by the ratio of the thermal gradient (*G*) to growth rate (*R*) at the solid/liquid interface. A higher *G/R* value is typically attained in the L-PBF process, forming a columnar morphology [89].

In order to differentiate between equiaxed and columnar grains, the aspect ratio of the grain shape was evaluated, as depicted in Figure 14a. This involved fitting an ellipse to each grain and determining the major and minor axes lengths of the ellipse to calculate the grain shape aspect ratio. For this purpose, columnar grains were identified as having a grain aspect ratio value of less than 0.3, while equiaxed grains had an aspect ratio value of higher than 0.4 [90]. Observing the data presented in Figure 14b,c, it can be inferred that the H-VED sample exhibited a slightly greater proportion of grain aspect ratio compared to the other two samples. This implies that the higher VED turns equiaxed grains into columnar grains. More specifically, looking at Figure 14c, it can be comprehended that the differences in grain aspect ratio are more predominant between 0.25 and 0.6, which further highlights the influence of VED on the solidification behavior of the austenite grains.

This study analysed the linkages between VED and sample texture of newly developed AISI 316L-Cu samples produced via the L-PBF process. However, it is crucial to emphasize that specimens exhibiting higher VED values demonstrated a crystal growth orientation that closely aligned with the BD, leading to an increased texture component and higher aspect ratios of the grains.

## 4. Conclusions

This research systematically examined the development of the new AISI 316L-Cu alloy via the L-PBF process and then studied in detail the correlations between the VED and the structural characteristics of this new alloy. The findings led to the following conclusions:

- The feasibility of in situ alloying of an AISI 316L-Cu alloy utilizing the laser powder bed fusion process from powder mixing was demonstrated.
- The optimum process parameters that can guarantee the lowest porosity fraction, as well as low surface roughness for the newly developed AISI 316L-Cu alloy, were reported.
- The research indicates that the surface roughness is significantly influenced by the VED, with a trend of decreasing surface roughness as VED increases until reaching an optimal surface condition. Subsequent increases in VED beyond this point are found to have a negative impact on the surface quality. Moreover, adjusting the VED values can lead to the fabrication of dense components, as evidenced by creating cubes with a density of 99.71%, achieved by applying a laser energy density of 101 J/mm^3^.
- Based on the XRD results, adding Cu in the present study did not influence the stability of the austenite phase of AISI 316L. The results suggest that increased values of VED lead to more opportunities for dislocation rearrangement and annihilation, resulting in reduced lattice distortion and a gradual increase in crystallite size.
- The microhardness results indicated that increases in porosity and grain size, and decreases in crystal defects led to a reduction in hardness. Moreover, the microhardness of AISI 316L-Cu alloys was higher than that of AISI 316L produced through L-PBF, primarily attributed to the enhanced solid solution strengthening effect resulting from the presence of Cu element.
- By integrating the findings from the XRD analysis and EBSD characterization, it can be inferred that increasing VED from L to M and H conditions tends to increase the grain size, reduce the proportion of low-angle boundaries, and decrease local microstrain and dislocation density.
- IPF maps revealed that 〈101〉 ∥ BD texture tends to increase from L to M and H conditions, as evidenced by the increase from 22% to 53% in the fraction of grains orienting at <101> in the BD. The ODF section analysis showed that the Goss texture was enhanced when moving from L to M and H conditions. However, all the samples possessed a strong Copper texture regardless of the conditions.

All in all, it can be concluded that, in the experimental phase to find the optimum process parameters for producing any new alloy by the L-PBF process, the VED value should be considered as a crucial factor, not only for determining the defect density but also the microstructural features.

## Figures and Tables

**Table 1:** Nominal chemical composition of the AISI 316L-Cu powder feedstock used in this research.

Elements	Cr	Ni	Mo	Cu	C	Mn	Si	P	S	Fe
AISI 316L-Cu (wt. %)	17.72	12.50	2.04	3.42	0.02	2.21	0.48	0.022	0.01	Bal.

**Table 2:** Process parameters used to produce different AISI 316L-Cu samples.

Sample No.	P [W]	h [mm]	v [mm/s]	t [mm]	VED [J·mm^−3^]
1	95	0.074	400	0.025	128.38
2		0.084	400		113.10
3		0.094	400		101.06
4		0.084	475		95.24
5		0.084	500		90.48
6		0.074	600		85.59
7		0.094	500		80.85
8		0.084	600		75.40

## Data Availability

The original contributions presented in the study are included in the article, further inquiries can be directed to the corresponding author.
